# Guidance on COVID-19 Vaccination in Hidradenitis Suppurativa Patients: A Modified Delphi Consensus of Experts

**DOI:** 10.1159/000521268

**Published:** 2022-01-10

**Authors:** Jonathan W. Rick, Devea R. De, Terri Shih, Afsaneh Alavi, Joslyn S. Kirby, Haley B. Naik, John W. Frew, Christopher J. Sayed, Jennifer L. Hsiao, Vivian Y. Shi

**Affiliations:** ^a^Department of Dermatology, University of Arkansas for Medical Sciences, Little Rock, Arkansas, USA; ^b^Jacobs School of Medicine and Biomedical Sciences, University at Buffalo, Buffalo, New York, USA; ^c^David Geffen School of Medicine, University of California, Los Angeles, California, USA; ^d^Department of Dermatology, Mayo Clinic, Rochester, Minnesota, USA; ^e^Department of Dermatology Hershey, Penn State Health Milton S. Hershey Medical Center, Hershey, Pennsylvania, USA; ^f^Department of Dermatology, University of California, San Francisco, San Francisco, California, USA; ^g^Department of Dermatology, Liverpool Hospital, Sydney, New South Wales, Australia; ^h^Department of Dermatology Chapel Hill, University of North Carolina at Chapel Hill, Chapel Hill, North Carolina, USA; ^i^Division of Dermatology, Department of Medicine, University of California Los Angeles, Los Angeles, California, USA

**Keywords:** Hidradenitis suppurativa, COVID-19, Vaccination, Guidelines, Expert consensus, Immunomodulators

## Abstract

**Introduction:**

Hidradenitis suppurativa (HS) patients may be at increased risk of COVID-19 infection and complications from their medications and comorbidities. There is a lack of expert consensus on recommendations for the COVID-19 vaccine for HS patients. Herein, we aim to provide expert-driven consensus recommendations regarding COVID-19 vaccinations in HS patients.

**Methods:**

A modified Delphi consensus survey developed by a core committee of 7 dermatologist HS experts consisting of 4 demographic questions and 12 practice statements was distributed to the US HS Foundation-sponsored provider listserv. Participants were attending physician HS experts. Survey results were to be reviewed by the core group and revised and resubmitted until consensus (≥70% agreement) was achieved.

**Results:**

Among the 33 survey participants, there were 30 (87%) dermatologists, 1 general surgeon, 1 plastic surgeon, and 1 rheumatologist. Consensus for all 12 statements on vaccine counseling and HS treatment counseling was achieved after the first round.

**Discussion/Conclusion:**

For now, this consensus can serve as a resource for clinicians discussing COVID-19 vaccination with their HS patients. These recommendations will need to be updated as new evidence on COVID-19 emerges.

## Introduction

Hidradenitis suppurativa (HS) is frequently associated with comorbidities and often requires immunomodulating therapies [1]. COVID-19 vaccination rates remain low in parts of the USA and worldwide due to vaccine hesitancy, misinformation, lack of guidance for practitioners, and lack of vaccine access [2]. Anecdotally, we have witnessed COVID-19 vaccine hesitancy and misinformation in the HS patient population. Recent studies examining COVID-19 outcomes among patients with and without HS have shown that HS patients, many of whom were on immunosuppressive and immunomodulating therapies, do not have worse outcomes than the general population [1, 3, 4]. Regardless of observed outcomes, vaccination remains a public health priority. We aim to provide expert consensus recommendations regarding COVID-19 vaccination in HS patients.

## Methods

**Fig. 1:**
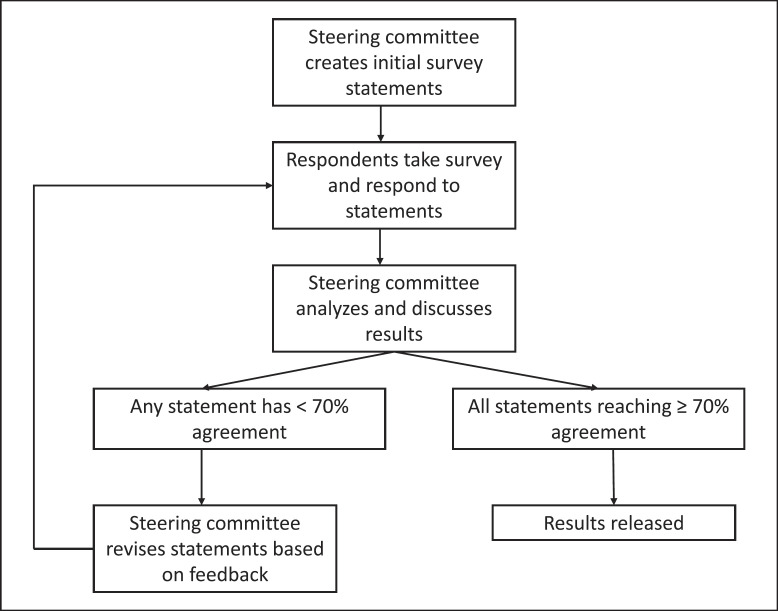
Modified Delphi consensus process.

We performed an anonymous RedCap modified Delphi survey of HS experts who are attending physicians and members of the US HS Foundation-affiliated provider listserv (Fig. 1). Practice statements were developed by a core committee of HS experts consisting of 7 board-certified dermatologists (6 practices in the USA, 1 in Australia). Participants answered 4 demographic questions and 12 practice statements (Table 1). Answer choices included: “agree without changes,” “agree with changes” and “disagree, please provide comment.” Survey results were reviewed and statements were to be revised and resubmitted until ≥70% consensus was achieved.

## Results

Among the 33 survey participants, there were 30 (87%) dermatologists, 1 general surgeon, 1 plastic surgeon, and 1 rheumatologist. In terms of patient volume, 12% of participants report seeing >50 HS patients per month, 48% report 26–50 per month, 24% report 11–25 per month, and 15% report 1–10 per month. Consensus was achieved after the first round. Practice statements are outlined in Table 1.

## Discussion

The consensus highlights that HS patients and their close contacts should receive the COVID-19 vaccination and follow preventative guidelines due to the potentially higher risk of COVID-19 complications. There should be no-to-minimal modification of HS treatment regimens, while patients receive their vaccination. HS patients should be reassured that there is currently no evidence to suggest that they have increased risk of vaccination-related complications or need routine antibody titer testing. Based on available data and our expert consensus, with minimal exception, patients should receive the COVID-19 vaccine without modifications to treatment regimen or vaccination timing [1].

Immunomodulatory and immunosuppressive medications may alter vaccine response and infection risks; however, further investigation is needed. Of note, recent guidelines from the Centers for Disease Control and Prevention recommend that patients on immunosuppressants and all biologics receive an additional dose of the vaccine [5]. Although there is no evidence to show that HS independently increases risk of poor outcomes from COVID-19 infection, comorbid conditions such as diabetes mellitus, obesity, and tobacco use may contribute to immune alteration [1, 6].

Ongoing studies, such as the University of California San Francisco led Global Hidradenitis Suppurativa COVID-19 Registry, will provide further data to guide recommendations in this patient population. Initial findings demonstrate that biologic therapies have not been associated with increased COVID-19 severity [7, 8].

## Conclusion

Many proposed statements currently lack data-driven evidence given limited available information on COVID-19 vaccination on HS patients. This consensus was performed prior to the recommendation of a third vaccine dose people who are moderately to severely immunocompromised [5]. Participants were limited to US experts, and these recommendations may not generalize to other countries.

Presently, this expert consensus can guide clinicians on COVID-19 vaccination in HS patients. These statements shall be updated appropriately as our understanding of COVID-19 and HS improves.

## Statement of Ethics

This study was reviewed and written informed consent was exempted by University of Arkansas for Medical Sciences Institutional Review Board (#262804).

## Conflict of Interest Statement

J.L.H. is on the board of directors for the HS Foundation (HSF) and is a consultant for Novartis and speaker for AbbVie. A.A. is consultant for Abbvie, BI, InflaRX, Janssen, Novartis, and UCB and investigator for BI and Processa. C.J.S. is on the board of directors of the HSF, has received research funding from Abbvie, Novartis, Incyte, InflaRx, Chemocentryx, and UCB, and has received honoraria for consulting and/or speaking from Abbvie, UCB, Novartis, and InflaRx. H.B.N. has received grant support from AbbVie, consulting fees from 23andme, Abbvie, and DAVA Oncology, advisory board fees from Boehringer Ingelheim, and is an investigator for Pfizer. She is also an Associate Editor for *JAMA Dermatology* and an unpaid board member of the US HSF. J.W.F. has conducted advisory work for Janssen, Boehringer Ingelheim, Pfizer, Kyowa Kirin, LEO Pharma, Regeneron, Chemocentryx, Abbvie, and UCB, participated in trials for Pfizer, UCB, Boehringer Ingelheim, Eli Lilly, CSL, Janssen, and received research support from Ortho Dermatologics and Sun Pharma. J.S.K. is consultant for Abbvie, Bayer, Incyte, InflaRX, Janssen, Novartis, and UCB; on the speaker bureau for AbbVie; and received research funding from Incyte. V.Y.S. is on the board of directors for the HSF, is a stock shareholder of Learn Health, and has served as an advisory board member, investigator, speaker, and/or received research funding from Sanofi Genzyme, Regeneron, AbbVie, Eli Lilly, Novartis, SUN Pharma, LEO Pharma, Pfizer, Incyte, Boehringer Ingelheim, Menlo Therapeutics, Dermira, Burt's Bees, Galderma, Kiniksa, UCB, TARGET-DERM, Altus Lab, MYOR, Polyfin, GpSkin, and Skin Actives Scientific.

## Funding Sources

No funding was received for this study.

## Author Contributions

All named authors met the International Committee of Medical Journal Editors (ICMJE) criteria for authorship for this manuscript and have given approval for submission.

## Data Availability Statement

All data generated or analyzed during this study are included in this article. Further enquiries can be directed to the corresponding author.

## Figures and Tables

**Table 1:** Expert consensus statements regarding COVID-19 vaccination for hidradenitis suppurativa patients

		Statements
1	VC	HS healthcare providers should engage HS patients in shared decision-making about receiving the COVID-19 vaccine

2	VC	HS patients with COVID-19 infection may have higher risk of poor outcomes compared to the general population due to HS, treatment-related factors, and common comorbidities (such as diabetes and obesity)

3	VC	Unless there are known allergies to vaccine components, HS patients (regardless of disease activity or severity) should receive the COVID-19 vaccine (with no preference for one vaccine over another) per government approval

4	VC	According to existing data, HS patients are NOT at increased risk of vaccine-related complications (e.g., thromboembolism) following COVID-19 vaccination compared to the general population

5	TC	HS patients on systemic immunomodulatory therapies may have a diminished response to COVID-19 vaccination compared to the general population

6	VC	HS patients should continue adherence to all preventative measures per public health guidelines after COVID-19 vaccination

7	TC	HS patients who are on the following medications (or any combination of the following medications): topical medications, systemic antibiotics, hormonal therapies, systemic retinoids, should receive the COVID-19 vaccine with no modifications to treatment regimen or vaccination timing

8	TC	HS patients on biologic agents (including TNF-alpha, IL-1, IL-12/23 or 23, IL-17 inhibitors) should receive the COVID-19 vaccine with no modifications to treatment regimen or vaccination timing

9	TC	HS patients on apremilast, prednisone (<20 mg/day), cyclosporine, or colchicine should receive the COVID-19 vaccine with no modifications to treatment regimen or vaccination timing

10	TC	HS patients on methotrexate should hold methotrexate 1 week after each vaccine dose

11	VC	Providers should not routinely order any lab testing (i.e., antibody titers) to assess COVID-19 immunity post-vaccination or in unvaccinated individuals

12	VC	Close contacts of HS patients should receive the COVID-19 vaccination whenever it is available to them to facilitate the protection of the HS patient

VC, vaccine counseling; TC, treatment counseling.
